# Correlation Between Tongue Morphology and Dental Arch Dimensions in Skeletal Class I and Class II Malocclusions: A Cephalometric Study

**DOI:** 10.7759/cureus.80946

**Published:** 2025-03-21

**Authors:** Saransha Mehra, Shelly Saxena, Saloni Pansotra, Jitendra Puri, Kashif Mohammed, Smriti Saha, Seema Gupta, Santosh Kumar

**Affiliations:** 1 Department of Orthodontics, Kothiwal Dental College and Research Centre, Moradabad, IND; 2 Department of Orthodontics, Teerthanker Mahaveer Dental College and Research Centre, Moradabad, IND; 3 Department of Orthodontics, Maharishi Markandeshwar College of Dental Sciences and Research, Mullana, IND; 4 Department of Orthodontics, Geetanjali Dental and Research Institute, Udaipur, IND

**Keywords:** dimensions, pattern, position, posture, skeletal, tongue, transverse

## Abstract

Introduction: Orthodontic stability depends on the balance between soft tissue forces and skeletal structures. The tongue, which is a key component of the oral musculature, influences the dental arch dimensions and occlusal relationships. An abnormal tongue posture is linked to malocclusion and altered craniofacial development. This study aimed to evaluate tongue morphology and its correlation with dental arch length (AL), arch width (AW), and palatal height (PH) in skeletal Class I and Class II patients.

Materials and methods: A prospective, observational cross-sectional study was conducted at the Department of Orthodontics from March 2022 to July 2024. A total of 80 subjects (40 Class I, 40 Class II) aged 18-30 years were included in the study. Cephalometric radiographs were obtained using a Carestream CS 8100 SC X-ray scanner (Carestream Dental, Atlanta, USA). Barium sulfate solution was applied to the tongue to enhance visibility. Tongue measurements, including tongue length (TL), tongue height (TH), posture, and position relative to the lower incisors and pharyngeal wall, were recorded. The transverse dimensions of the dental arch were measured using digital callipers on the dental casts. Intra-observer reliability was assessed using the intraclass correlation coefficient (ICC). Data analysis was performed using the Mann-Whitney U test and correlation analysis.

Results: Skeletal Class I patients had significantly greater TH, decreased TL, and higher tongue posture than Class II patients. In contrast, Class II individuals exhibited lower tongue posture, increased TL, a greater distance between the tongue and lower incisors, and a reduced distance to the pharyngeal wall. Class I patients also had significantly greater maxillary AL and AW, whereas Class II patients demonstrated deeper palatal vaults. Correlation analysis revealed that tongue morphometric variations influenced AL and AW across both skeletal patterns, with the tongue position affecting maxillary constriction in Class II patients.

Conclusion: Class II patients exhibited a lower tongue posture, increased TL, and deeper palatal vaults, which may contribute to maxillary constriction and malocclusion. These findings emphasize the role of tongue posture in arch development and the need for myofunctional therapy in Class II treatment. Future studies using three-dimensional imaging are recommended.

## Introduction

Orthodontics is a complex field that involves the study of hard- and soft-tissue structures. However, understanding the balance between these forces and the oral musculature remains limited [1]. Many orthodontic treatment approaches prioritize camouflage techniques to achieve dental correction of malocclusions, but failure to achieve neuromuscular equilibrium can lead to relapse in most cases [2]. The balance between the labiolingual muscular forces and tongue muscles is crucial for maintaining the stability of the arch shape and tooth position.

The tongue, the largest component of the oral cavity, plays a significant role in shaping various skeletal malocclusions. Abnormalities in tongue functions, such as mastication, deglutination, speech, and breathing, can be directly correlated with the development of malocclusions and speech defects [3]. These functional abnormalities can occur at any age but are more common in children. Variations in the size of the tongue can be correlated with genetic and racial makeup, while malocclusion itself may contribute to abnormal tongue posture [4].

Achieving a stable, functional, and aesthetically pleasing arch form is essential for orthodontists to anticipate the treatment outcomes. Factors such as genetic influences, bone growth, tooth eruption and inclination, external forces, functional adaptations, and ancestral background contribute to the formation of dental arches [5]. Resting muscle pressure may influence the dental arch form and tooth positioning, as the tongue remains in a resting position for most of the time, significantly impacting dentoskeletal development [3].

The forces from the tongue have been proposed as a potential link with the transverse dimension of the dental arches [3,5,6]. Bourdiol et al. suggested that palatal vault height is directly associated with tongue posture and the size [7]. Therefore, understanding the correlation between tongue morphology and dental structures can provide valuable insights into orthodontic diagnosis and treatment planning, especially in patients with different skeletal malocclusions.

Previous research has explored the relationship between tongue posture and dental arch development [8]; however, few studies have quantitatively evaluated tongue morphology using lateral cephalometric radiographs [9]. Lateral cephalometry provides a reliable means of assessing craniofacial structures in both skeletal and dental contexts, allowing for precise measurements of tongue dimensions and their association with the dental arch and palatal parameters [8]. This study aimed to evaluate tongue morphology and its correlation with dental arch length (AL), dental arch width (AW), and palatal height (PH) in skeletal Class I and Class II subjects. Specifically, this study aimed to measure key tongue parameters, including tongue length (TL), tongue height (TH), tongue posture, and distance from the pharyngeal wall and lower incisors in patients with Class I and Class II skeletal patterns. Furthermore, this study aimed to analyze the relationship between tongue morphology and dental arch dimensions by correlating tongue measurements with the mandibular AL and AW in both classifications. Additionally, the study assessed the correlation between tongue morphology and transverse arch dimensions, including AL, AW, and PH, to better understand the influence of tongue anatomy on craniofacial structures.

## Materials and methods

Study design and setting

This prospective, observational, cross-sectional study was conducted at the Department of Orthodontics, Kothiwal Dental College and Research Centre, Moradabad, India, from May 2023 to October 2024. The study was initiated after obtaining ethical clearance from the institutional ethics committee (KDCRC/IERB/04/2023/29) and followed all the principles of the Declaration of Helsinki. All patients were informed of the study, and written informed consent was obtained. The cephalometric scans used in this study were part of the diagnostic records for orthodontic treatment.

Sample size estimation

G*Power software version 3.2.9 (Heinrich Heine University Düsseldorf, Düsseldorf, Germany) was used for the sample size calculation. Based on the calculated effect size of 0.641 from a previous study [10], 5% level of precision, 95% confidence level, and 80% power of the study, the minimum sample size was calculated to be 80.

Eligibility criteria

The study involved 80 subjects aged 18-30 years with skeletal Class l malocclusion (an ANB angle of 2°-4°) and skeletal Class II (ANB angle >4°) with Angle's Class II Division 1 malocclusion, with no history of previous orthodontic treatment and no history of any surgical procedure of the tongue. Those with a history of previous tongue deformity, missing teeth except the third molar, Class III malocclusion with underlying Class III skeletal base, pregnant and lactating females, patients with neuromuscular and psychological problems, and patients with any lesion affecting the tongue were excluded.

Methodology

**Figure 1:**
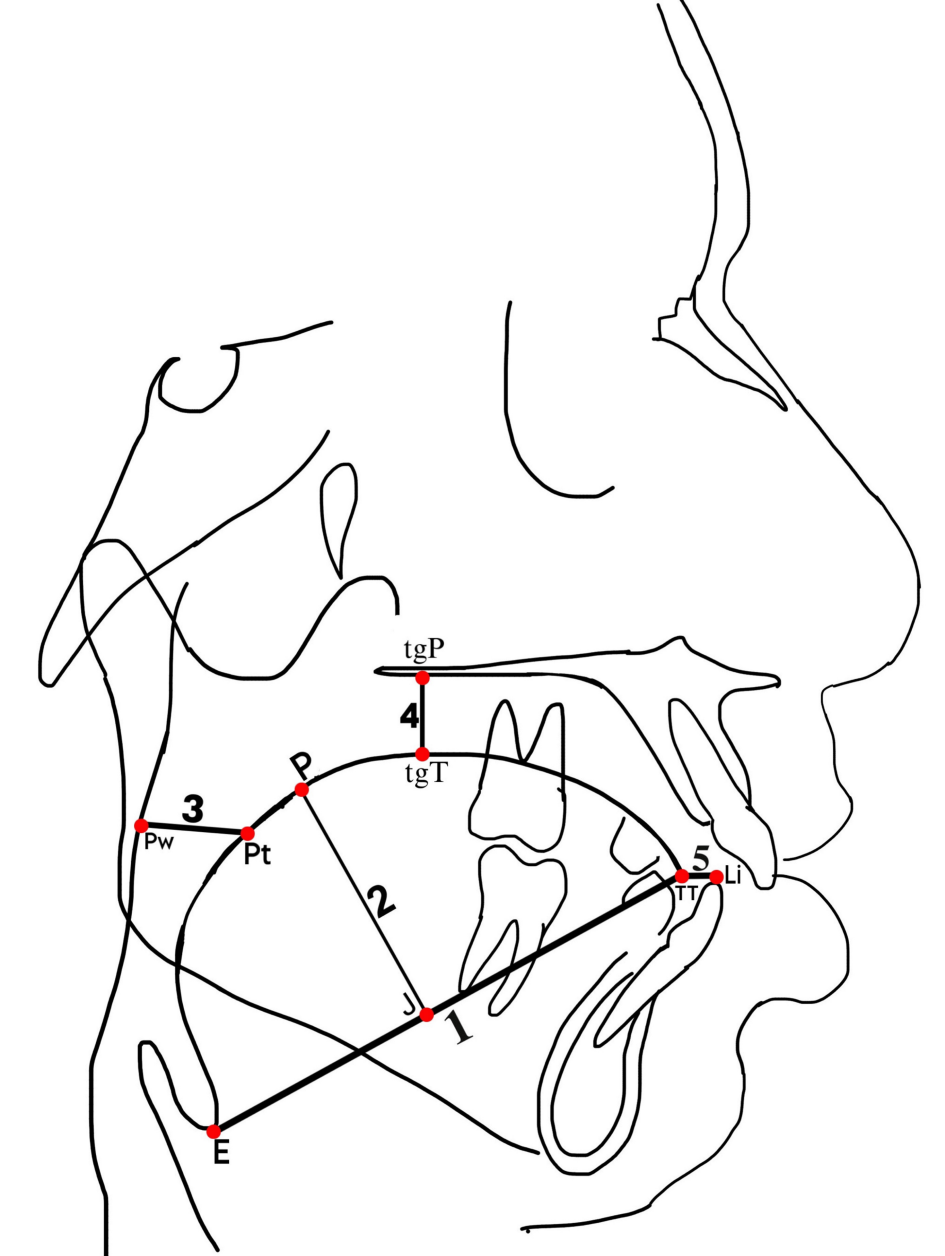
Tongue morphometrics on the lateral cephalogram: tongue length from the epiglottis point (E) to the tongue tip (TT) point is represented by 1, tongue height from the dorsum of the tongue (P) to the midpoint of the E-TT line (J) point is represented by 2, distance of the tongue from the pharyngeal wall (Pw) to the point on contour of the tongue (Pt) is represented by 3, tongue posture from the intersection point at the palatal contour (tgP) to the intersection point at the dorsum of the tongue (tgT) is represented by 4, and distance of the tongue from lower incisors (Li), from TT to the Li point, is represented by 5. This figure was created by the author.

Patients were divided into two groups: Group 1 (n = 40) with skeletal Class I patients and Group 2 (n = 40) with skeletal Class II patients. Radiographs were obtained using a Carestream CS 8100 SC X-ray machine (Carestream Dental, Atlanta, USA) at the Department of Oral Medicine and Radiology. A radiographically opaque solution of barium sulfate was applied to the dorsum of the patient’s tongue, enhancing the visibility of the tongue's soft tissue outline on X-rays. All cephalograms were traced on a standard 8x10-inch acetate paper with a thickness of 0.003 inch using standard techniques and a 3H pencil while illuminated by a view box. The tracings were performed by a single operator in a consistent manner to minimize errors caused by inter-operator variability. The cephalometric landmarks, reference planes, and linear and angular measurements recorded in this study are shown in Figure 1, Table 1, and Table 2.

**Table 1:** Landmarks used in the study

S. no.	Landmarks	Description
1	ANS	Anterior nasal spine (ANS) is the most prominent tip of the sharp bony process of the maxilla at the lower margin of anterior nasal opening
2	E	The lowest and most prominent point on epiglottis
3	Li	The tip of the most prominent mandibular incisors
4	Mc	The point on the cervical, and distal third of the last erupted permanent molar
6	mc	The tip of the distobuccal cusp of the maxillary first permanent molar
7	U	Most anterior point on uvula
8	O	Middle of the linear distance between U and Li projected on the mc-Li line
9	Pt	Intersection point of the occlusal plane with the contour of the tongue
10	Pw	Intersection point of the occlusal plane with the pharyngeal wall
10	TT	Tongue tip
11	N	Nasion is most anterior point on the frontonasal suture in the midsagittal plane
13	Point A	Most posterior midline point in the concavity between the ANS and the prosthion
14	Point B	Most posterior midline point in the concavity between the most superior point on the alveolar bone overlying the lower incisors and pogonion
15	ANB angle	The angle between the NA line and NB line
16	tg4 line	Line constructed on O at 90° to the mc-Li line
20	tgT	The intersection point between the dorsum of the tongue and tg4 line
21	tgP	The intersection point between the palatal contour and tg4 line
22	Occlusal plane	Occlusal plane is formed by joining midpoints of the overlap of mesiobuccal cusps of first molars and the buccal cusps of the first premolars
26	E-TT line	The line between the most antero-inferior point of the epiglottis and the tongue tip
27	Point J	Midpoint of the E-TT line
28	Point P	Perpendicular dropped to the E-TT line from point J to the upper border of the dorsum of the tongue

**Table 2:** Parameters for the assessment of tongue morphometrics TT: tip of the tongue, Li: lower incisor

S. no.	Parameters	Description
1.	Tongue length (mm)	Measured as the linear distance of the E-TT line.
2.	Tongue height (mm)	Tongue height (TH) indicates the size of the tongue; measured as the linear distance between point J and P.
3.	Distance from the tongue to the pharyngeal wall (mm)	Measured as the linear distance between point Pt and Pw on the occlusal plane.
4.	Tongue posture (mm)	Measured as the linear distance between point tgT and tgP.
5	Distance from the tongue to lower incisors (mm)	Measured as the linear distance between TT and Li.

**Figure 2:**
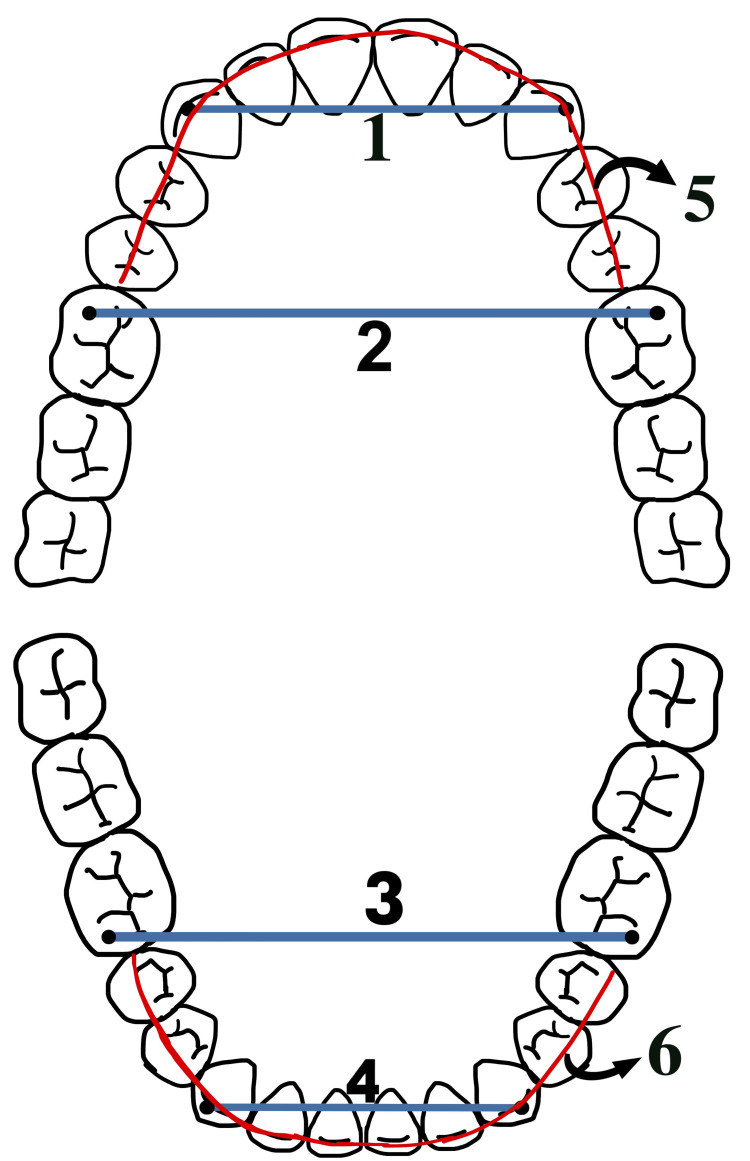
Measurements of the arch width and arch length in the maxillary and mandibular arch: the maxillary intercanine width is represented by 1, the maxillary intermolar width is represented by 2, the mandibular intermolar width is represented by 3, the mandibular intercanine width is represented by 4, the maxillary arch length is represented by 5, the mandibular arch length is represented by 6 This figure was created by the author.

**Figure 3:**
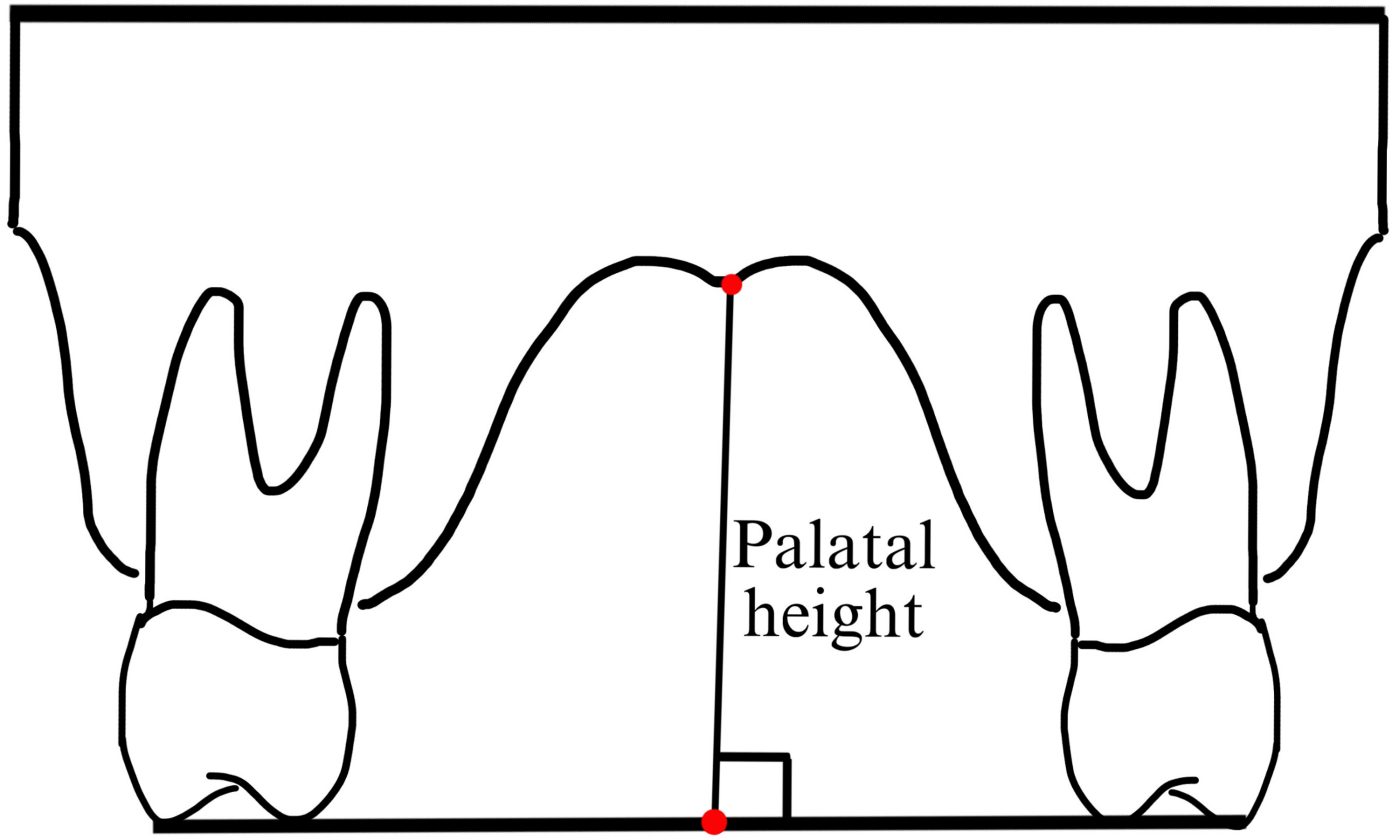
Measurement of the palatal height in the maxillary arch This figure was created by the author.

Model analysis was performed on dental casts of the patients, where distinct impressions of the maxillary and mandibular arches were taken using alginate as an elastomeric, irreversible hydrocolloid impression material. Dental casts were formed by pouring impressions using a dental stone material, and a universal digital calliper was used to measure the transverse widths of the upper and lower dental casts. The results are shown in Figure 2, Figure 3, and Table 3.

**Table 3:** Parameters for the assessment of transverse dimensions of maxillary and mandibular arches

S. no.	Parameters	Description
1	Maxillary intercanine width (mm)	It was measured between cusp tips of maxillary canines.
2	Mandibular intercanine width (mm)	It was measured between cusp tips of mandibular canines.
3	Maxillary intermolar width (mm)	It was measured between mesiobuccal cusp tips of maxillary first permanent molars.
4	Mandibular intermolar width (mm)	It was measured between mesiobuccal cusp tips of mandibular first permanent molars.
5	Maxillary arch length (mm)	It was measured using a brass wire from the mesial surface of the first maxillary molar on the right side to the mesial surface of the first maxillary molar on the left side.
6	Mandibular arch length (mm)	It was measured using a brass wire from the mesial surface of the first mandibular molar on the right side to the mesial surface of the first mandibular molar on the left side.
7	Palatal height (mm)	It was measured by dropping a perpendicular from the line connecting the occlusal surfaces of the maxillary first molar teeth to the midpalatal surface.

Reliability testing

Tests were conducted to assess intra-observer reliability, which was assessed by repeating measurements on 20 pre-treatment cephalometric radiographs and their corresponding maxillary and mandibular models. The intraclass correlation coefficient (ICC) was calculated to assess the consistency between measurements.

Statistical analysis

Statistical analysis was performed utilizing IBM SPSS Statistics for Windows, version 23.0 (IBM Corp., Armonk, NY). The normality of data distribution was examined using the Shapiro-Wilk test and further validated by a Q-Q plot, which revealed a non-normal distribution. Intraclass correlation was used to ensure the reliability of the measured data. Intergroup comparisons of tongue and jaw morphometric measurements in skeletal Class I and Class II patients were carried out using the Mann-Whitney U test. Statistical significance was established at p < 0.05, with Bonferroni correction applied to account for multiple comparisons and reduce the risk of Type 1 errors.

## Results

The results showed consistently high intra-observer reliability (r) in the range of 0.85-0.89. These reliability measures highlight the consistency of the study's methods and underscore the reliability of the data collected, which is crucial for ensuring valid conclusions regarding the differences in morphometric parameters across groups. The independent t-test results indicated no noteworthy variation in age among sexes within either the Class I or Class II group. The mean age for female patients was 19.75 ± 2.56 years, while for male patients was 20.1 ± 3.21 years. This confirms that the age distribution was comparable across sexes and malocclusion classes, eliminating age as a confounding factor.

The Mann-Whitney U test demonstrated noteworthy differences in all tongue morphometric parameters between the two malocclusion groups. Skeletal Class I patients had a statistically significantly greater TH, decreased TL, and higher tongue posture, and the tongue was found to be closer to the lower incisors and away from the pharyngeal wall, compared to skeletal Class II patients (Table 4).

**Table 4:** Comparison of tongue morphometrics (in mm) in different malocclusions using the Mann-Whitney U test CI: confidence interval, TT: tip of the tongue, Li: lower incisor, Pw: pharyngeal wall Data is presented in the form of means and standard deviations. *p < 0.05, significant.

Parameters	Groups	N	Mean	Standard deviation	CI at 95% of mean	U value	p value
Tongue height	Class I	40	28.462	2.418	27.69-29.24	1322.5	0.016*
	Class II	40	25.300	2.759	24.42-26.18		
Tongue length	Class I	40	65.457	3.789	64.25-66.67	1252	0.022*
	Class II	40	69.275	2.650	68.43-70.12		
Tongue posture	Class I	40	4.128	0.478	3.97-4.28	16	0.015*
	Class II	40	6.048	0.600	5.86-6.24		
Distance TT to Li	Class I	40	4.388	1.043	4.05-4.72	1493	0.021*
	Class II	40	7.438	0.603	7.24-7.63		
Distance of tongue from Pw	Class I	40	19.017	0.744	18.78-19.26	1498	0.001*
	Class II	40	16.295	1.457	15.83-16.76		

The comparison of transverse dimensions between the groups revealed that skeletal Class I patients had significantly greater maxillary intercanine and intermolar AW and maxillary AL than skeletal Class II patients did. However, PH was significantly higher in patients with skeletal Class II pattern (Table 5).

**Table 5:** Comparison of transverse dimensions (in mm) in different malocclusions using the Mann-Whitney U test CI: confidence interval Data is presented in the form of means and standard deviations. *p < 0.05, significant.

Parameter	Group	N	Mean	Standard deviation	CI at 95% of mean	U value	p value
Maxillary intercanine width	Class I	40	31.41	2.024	30.76-32.06	1340.5	0.024*
	Class II	40	27.60	1.958	26.97-28.23		
Mandibular intercanine width	Class I	40	25.72	1.189	24.34-25.10	1184.0	0.732
	Class II	40	23.14	1.167	22.77-23.51		
Maxillary intermolar width	Class I	40	53.42	2.847	52.51-54.34	1139.0	0.001*
	Class II	40	51.08	2.399	50.32-51.86		
Mandibular intermolar width	Class I	40	45.86	2.321	45.12-46.60	1104.5	0.186
	Class II	40	44.13	2.152	43.45-44.83		
Maxillary arch length	Class I	40	71.19	1.872	70.59-71.79	1213.5	0.045*
	Class II	40	69.88	2.667	69.03-70.74		
Mandibular arch length	Class I	40	65.76	4.060	64.47-67.07	111.0	0.248
	Class II	40	62.72	1.765	62.16-63.28		
Palatal height	Class I	40	18.21	0.706	17.99-18.44	4.0	0.001*
	Class II	40	20.78	1.296	20.37-21.19		

The Mann-Whitney U test revealed significant sex-specific differences in tongue morphometric parameters between the two groups. Both male and female patients with skeletal Class I malocclusions had a statistically greater TH, whereas both the male and female patients had a statistically greater TL, lower tongue posture, and increased distance from the lower incisors in the skeletal Class II group (Table 6).

**Table 6:** Sex-wise comparison of tongue morphometrics (in mm) in malocclusions using the Mann-Whitney U test CI: confidence interval, M: male, F: female, TT: tip of the tongue, Li: lower incisor, Pw: pharyngeal wall Data is presented in the form of means and standard deviations. *p < 0.05, significant.

Parameter	Gender	Group	Mean	Standard deviation	95% CI for mean	p value
Tongue height	F	Class I	26.98	2.07	26.01-27.95	0.023*
		Class II	24.53	3.69	22.80-26.26	
	M	Class I	29.95	1.76	29.12-30.77	0.025*
		Class II	26.07	0.88	25.66-26.48	
Tongue length	F	Class I	64.89	3.40	63.30-66.48	0.034*
		Class II	67.90	0.54	67.65-68.15	
	M	Class I	66.03	4.15	64.08-67.97	0.002*
		Class II	70.65	3.18	69.16-72.14	
Tongue posture	F	Class I	3.93	0.40	3.74-4.12	0.004*
		Class II	5.74	0.55	5.48-6.00	
	M	Class I	4.33	0.47	4.10-4.55	0.045*
		Class II	6.36	0.49	6.13-6.58	
Distance TT to Li	F	Class I	3.49	0.37	3.32-3.66	0.021*
		Class II	7.06	0.47	6.84-7.28	
	M	Class I	5.29	0.63	4.99-5.58	0.014*
		Class II	7.82	0.47	7.59-8.04	
Distance of tongue from Pw	F	Class I	18.79	0.70	18.47-19.12	0.04
		Class II	15.93	1.79	15.09-16.77	
	M	Class I	19.24	0.74	18.90-19.58	0.02
		Class II	16.66	0.94	16.22-17.1	

The Mann-Whitney U test revealed significant sex-specific differences in transverse dimensions between the two groups. Both males and female patients with skeletal Class I malocclusions had a statistically greater maxillary and mandibular intercanine and intermolar AW; however, the difference was not statistically significant for mandibular intermolar AW in females. The maxillary and mandibular ALs were also greater in skeletal Class I patients of both sexes. Skeletal Class II patients exhibited deep palatal vaults compared to skeletal Class I patients of both sexes (Table 7).

**Table 7:** Sex-wise comparison of jaw morphometrics (in mm) in malocclusions using the Mann-Whitney U test CI: confidence interval, M: male, F: female Data is presented in the form of means and standard deviations. *p < 0.05, significant.

Parameter	Gender	Group	Mean	Standard deviation	95% CI for mean	p value
Maxillary intercanine width	F	Class I	30.03	1.00	29.56-30.50	0.001*
		Class II	26.10	1.54	25.37-26.82	
	M	Class I	32.79	1.84	31.93-33.65	0.001*
		Class II	29.11	0.85	28.71-29.50	
Mandibular intercanine width	F	Class I	24.21	0.99	23.74-24.68	0.001*
		Class II	22.30	0.93	21.86-22.74	
	M	Class I	25.23	1.17	24.68-25.78	0.001*
		Class II	23.98	0.66	23.67-24.29	
Maxillary intermolar width	F	Class I	52.36	3.04	50.94-53.79	0.024*
		Class II	50.05	2.67	48.8-51.29	
	M	Class I	54.49	2.25	53.43-55.54	0.001*
		Class II	52.13	1.55	51.40-52.86	
Mandibular intermolar width	F	Class I	45.08	2.23	44.03-46.13	0.086
		Class II	43.75	2.79	42.44-45.06	
	M	Class I	46.65	2.18	45.62-47.67	0.001*
		Class II	44.53	1.19	43.97-45.08	
Maxillary arch length	F	Class I	70.08	1.74	69.26-70.89	0.046*
		Class II	68.86	0.62	68.57-69.16	
	M	Class I	72.31	1.24	71.73-72.89	0.001*
		Class II	70.91	3.47	69.28-72.53	
Mandibular arch length	F	Class I	64.22	4.29	62.21-66.23	0.022*
		Class II	61.31	1.24	60.73-61.89	
	M	Class I	67.32	3.23	65.80-68.83	0.042*
		Class II	64.13	0.82	63.75-64.51	
Palatal height	F	Class I	17.70	0.46	17.48-17.91	0.001*
		Class II	20.47	1.38	19.83-21.11	
	M	Class I	18.74	0.49	18.51-18.97	0.001*
		Class II	21.09	1.16	20.55-21.63	

In the mandibular arch, for skeletal Class I patients, the intercanine AW showed a moderately significant correlation with TH, and AL showed a significant correlation with TL, tongue posture, and tongue distance from the lower incisors. In skeletal Class II patients, the intercanine AW showed a positive correlation with TH and with the distance of the tongue from the lower incisors. The intermolar AW showed a positive correlation with TH; AL showed a positive correlation with TH, tongue posture, and the distance of the tongue from the lower incisors. In the mandibular arch, for skeletal Class I patients, the intercanine and intermolar AW showed a positive correlation with TH, TL, and the distance of the tongue from the lower incisors. In skeletal Class II patients, intercanine AW showed a positive correlation with TH, TL, and tongue distance from the lower incisors. Intermolar AW showed a positive correlation with TH, while AL showed a positive correlation with TH, TL, and tongue posture (Table 8).

**Table 8:** Correlation between tongue morphometrics and transverse dimensions using the Spearman correlation test TT: tip of the tongue, Li: lower incisor, Pw: pharyngeal wall, IC: intercanine width, IM: intermolar width, AL: arch length Very weak correlation: 0.0<∣r∣<0.20; weak: 0.2≤∣r∣<0.4; moderate: 0.4≤∣r∣<0.6 *p < 0.05, significant

Parameters		Mandible						Maxilla					
		Class I			Class II			Class I			Class II		
		IC	IM	AL	IC	IM	AL	IC	IM	AL	IC	IM	AL
Tongue height	r	0.40	0.23	0.02	0.32	0.37	0.53	0.51	0.34	0.22	0.41	0.42	0.42
	p	0.01*	0.146	0.885	0.043*	0.019*	.001*	0.001*	0.032*	0.176	0.009*	0.007*	0.008*
Tongue length	r	0.20	0.22	0.34	-0.01	0.05	0.08	0.56	0.49	0.41	0.29	-0.02	0.36
	p	0.22	0.169	0.034*	0.94	0.739	0.618	0.001*	0.001*	0.009*	0.065	0.909	0.024*
Tongue posture	r	0.19	0.17	0.37	0.29	0.24	0.36	0.36	0.24	0.21	0.45	0.21	0.32
	p	0.252	0.301	0.018*	0.070	0.143	0.022*	0.022*	0.137	0.196	0.004*	0.189	0.044*
TT to Li distance	r	0.29	0.36	0.38	0.53	0.19	0.52	0.59	0.38	0.36	0.62	0.25	0.09
	p	0.067	0.024*	0.016*	0.001*	0.234	0.001*	0.001*	0.016*	0.022*	0.001*	0.127	0.593
Tongue distance from Pw	r	0.02	-0.12	0.27	0.08	-0.14	-0.04	0.05	0.14	0.24	0.26	-0.01	0.02
	p	0.926	0.446	0.083	0.608	0.384	0.786	0.779	0.383	0.123	0.099	0.923	0.897

## Discussion

Tongue morphology plays a crucial role in shaping the oral cavity, influencing dental arch dimensions, and occlusal relationships [3]. This study aimed to evaluate tongue morphology and its correlation with dental AL, AW, and PH in skeletal Class I and Class II patients. Lateral cephalometry was used in this study because of its accessibility and cost-effectiveness, compared to cone-beam computed tomography (CBCT). Barium sulfate was used to enhance tongue visibility in radiographs, improve contrast, and enable accurate measurement of tongue position and morphology. Dental casts were used for measurement because they provided a precise and stable representation of the dentition and oral structures, allowing for the accurate assessment of dental AL, AW, and PH. The inclusion criteria of 18-30 years ensured stable tongue morphology, eliminating age as a confounding factor, and enhancing the validity of the findings by attributing differences to structural variations rather than demographic influences.

Tongue morphometric parameters of tongue size between both malocclusion groups revealed that skeletal Class I subjects exhibited a higher tongue position; this higher tongue position may contribute to a balanced occlusal relationship, as the tongue exerts forces that help maintain dental arch stability and width. Conversely, reduced TH in Class II could indicate lower tongue posture, which is supported by findings from previous studies by Abu Allhaija et al. [11] and Shinde et al. [8].

TL was notably greater in Class II patients than in Class I patients, consistent with the findings of Deshkar et al. [3]. In Class I malocclusions, the relationship between the upper and lower jaws is more aligned, allowing the tongue to rest in a neutral position within the oral cavity. Additionally, in Class I, the upper portion of the tongue remains in close contact with the palate, aiding normal speech, swallowing, and dental arch stability without requiring adaptive elongation.

In Class II malocclusions, a pronounced overjet from a prognathic maxilla, retrognathic mandible, or both can lead to alterations in tongue posture and function. In cases of maxillary prognathism, the tongue may adopt a more anterior position, potentially affecting speech function. Insufficient tongue pressure on the palate can affect palatal morphology, often resulting in a high-arched and narrow palate [12]. Lower tongue posture in Class II individuals may be attributed to a retrognathic mandible, which alters the functional space available for the tongue. The decreased TH and increased TL in Class II patients might reflect an adaptive mechanism to accommodate constrained oral cavity dimensions [13,14]. The positioning of the tongue closer to the pharyngeal wall in Class II patients may also contribute to differences in airway morphology and function, which could have clinical implications in orthodontic treatment planning. Patients with skeletal Class II malocclusions often exhibit retrognathic mandibles, which can push the tongue downward and backward into the oropharynx, reducing the pharyngeal airway [15].

Lower tongue posture observed in skeletal Class II patients can exacerbate maxillary constriction and malocclusion. This could have been a reason for the decreased transverse dimensions of the maxillary arch and deep palatal vault in skeletal Class II patients [16]. In individuals with normal tongue posture (resting against the palate), the tongue provides continuous, gentle pressure on the maxillary arch, promoting transverse development [17]. However, in skeletal Class II patients with lower tongue posture, this expansive force is reduced or absent, leading to insufficient maxillary width development and a constricted arch. With low tongue posture, the balance between the intraoral and extraoral muscular forces is disrupted. The buccinator (cheek muscles) and orbicularis oris (lip muscles) exert unopposed inward pressure on the maxilla, contributing to its constriction and leading to a high-arched (deep palatal vault) and narrow palate [16].

The correlation analysis in this study further revealed that variations in tongue morphology can influence AW and AL, particularly in patients with different skeletal classifications. The positioning and size of the tongue can exert pressure on the dental arches, potentially influencing arch form and dimensions. Understanding these correlations can help clinicians develop targeted interventions to optimize oral function and improve orthodontic outcomes. Similar findings were reported by Yu et al. [18], who found a positional correlation between tongue pressure and arch dimensions, such as AL and AW.

Clinical implications of the study

The findings of this study have important clinical implications in orthodontic diagnosis and treatment planning. Recognizing the differences in tongue morphology and arch dimensions between skeletal Class I and II patients can help guide individualized treatment approaches. Class II patients, characterized by lower tongue posture and a deeper palatal vault, may benefit from myofunctional therapy to train proper tongue positioning or palatal expansion techniques to improve transverse maxillary dimensions. As tongue posture significantly influences dental arch development, orthodontic treatment should incorporate strategies to optimize tongue positioning. Appliances such as tongue cribs and myofunctional exercises can be effective in promoting correct tongue posture, thereby enhancing arch stability and alignment. Another critical factor is the potential impact of tongue posture and palatal morphology on the airway function. Class II patients with deep palatal vaults may be more prone to airway compromise, necessitating thorough evaluation and appropriate intervention to improve airway patency. By integrating these considerations into treatment planning, clinicians can optimize orthodontic outcomes, improve functional stability, and enhance overall patient health.

Limitations

This study had several limitations. First, it relies on lateral cephalometric radiographs, which provide two-dimensional images and may not fully capture the three-dimensional complexity of tongue morphology and dental arches. The use of barium sulfate to enhance tongue visibility may introduce variability in the measurement accuracy. Additionally, the study had a limited sample size, was conducted at a single institution, and did not take skeletal Class III and dental Class II Division 2 patients, which may affect the generalizability of the findings. Gender-based variations were considered, but other factors such as ethnicity and genetic influences were not explored. The present study did not measure the forces of the tongue, an important factor in determining the transverse dimensions of the dental arches. Therefore, future studies using three-dimensional imaging techniques and electromyographic examination of the tongue on a larger and more diverse sample are needed to validate these findings.

## Conclusions

This study highlights significant differences in tongue morphology and dental arch dimensions between skeletal Class I and Class II patients. Class II patients exhibited lower tongue posture, an increased tongue length, and a deeper palatal vault, which may contribute to maxillary constriction and malocclusion. These findings underscore the role of tongue posture in arch development and the need for incorporating myofunctional therapy and palatal expansion in Class II treatment plans. Additionally, a positive correlation was observed between tongue morphometrics and arch dimensions. The proximity of the tongue to the pharyngeal wall in Class II patients emphasizes the potential impact of tongue posture on airway function. Future research using advanced imaging techniques is recommended to further explore these associations and to enhance treatment strategies.
